# Opportunities and challenges in the application of spatiotemporal transcriptomics in plant research

**DOI:** 10.3389/fpls.2025.1684057

**Published:** 2025-10-15

**Authors:** Peilei Deng, Jiaruo Huang, Wencan He, Zhiyuan Li, Cun Guo, Guoxin Chen, Xiaoxu Li, Kejun Zhong, Wei Luo, Bo Kong

**Affiliations:** ^1^ Technology Center, China Tobacco Hunan Industrial Co., Ltd., Changsha, China; ^2^ Beijing Life Science Academy, Beijing, China; ^3^ Graduate School of Kookmin University, Seoul, Republic of Korea; ^4^ Tobacco Research Institute, Chinese Academy of Agricultural Sciences, Qingdao, China; ^5^ College of Agriculture, Tarim University, Alaer, China

**Keywords:** spatiotemporal transcriptomics, plant research, bioinformatics approaches, multi-omics and computational biology, plant biology

## Abstract

Spatiotemporal heterogeneity is recognized as a key driver of functional diversity in tissues. Spatial transcriptomics, which integrates high-throughput transcriptomics with high-resolution tissue imaging, enables the precise mapping of gene expression patterns at the tissue section level. This technology overcomes the limitations of traditional transcriptomics by providing spatial context and applying unbiased bioinformatics approaches. With the rapid advancement of sequencing technologies, spatial transcriptomics is a pivotal tool for exploring cell fate determination, tissue development, and disease mechanisms, and its underlying principles, technical variations, practical performance, and future directions collectively provide robust theoretical and methodological support for systematically unveiling the spatiotemporal regulation of life processes.

## 1 Introduction

**Figure 1:**
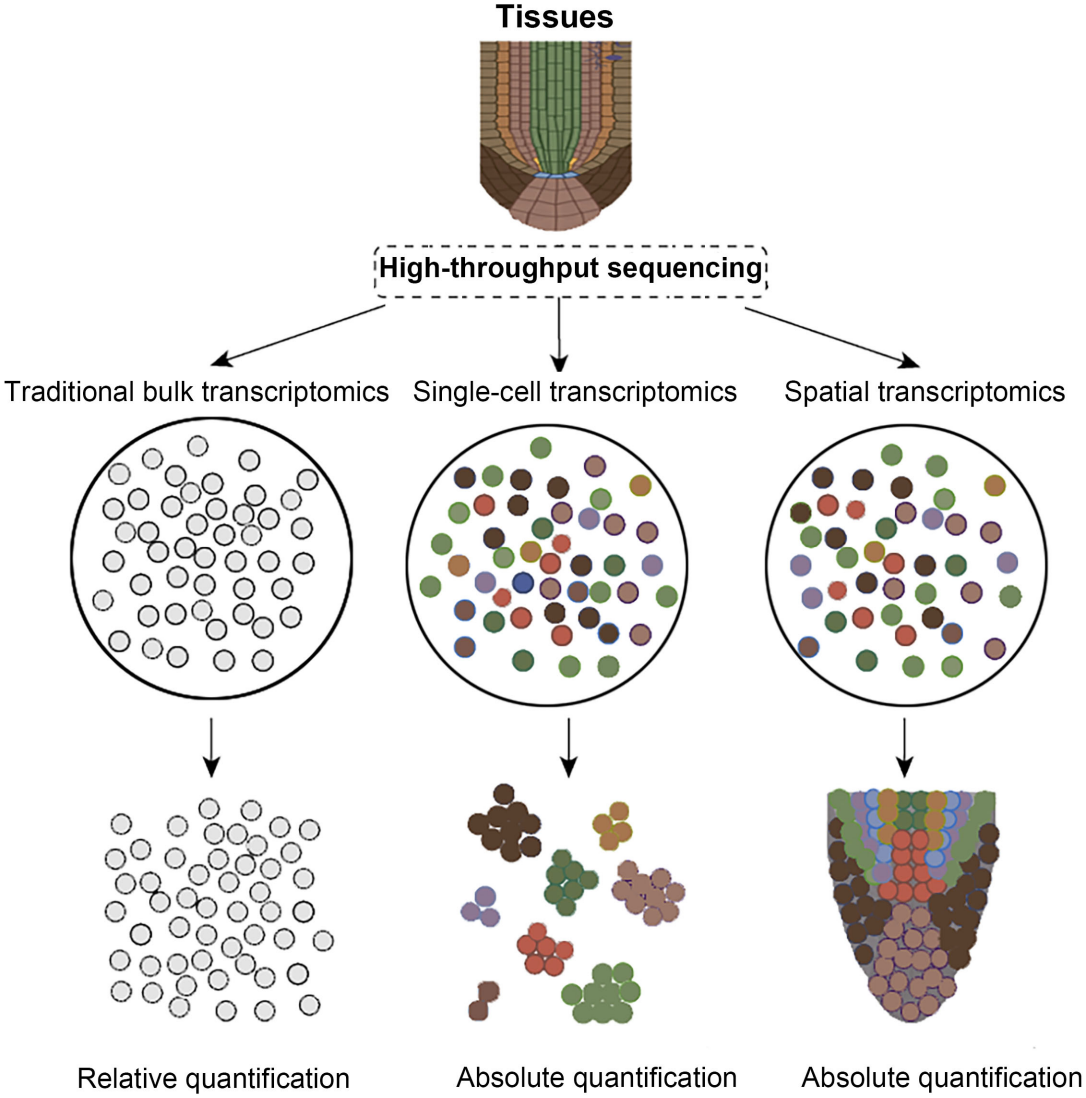
Comparison of transcriptomic technologies. Colored circles indicate distinct cell types, whereas gray circles show that RNA-seq cannot differentiate them.

As the fundamental structural and functional units of organisms, cells display profound spatiotemporal heterogeneity across developmental stages, spatial locations, and microenvironments, rendering the dissection of intricate transcriptional regulatory networks within multicellular systems a central challenge in modern life-science research. Traditional bulk RNA sequencing, which analyzes whole tissues or organs, can only obtain averaged gene expression levels, making it difficult to reveal rare cell subpopulations and their subtle gene expression differences (**Figure 1**) (Jiang et al., 2022; Li et al., 2022; Cao et al., 2024a, 2024b, 2025; Jiang et al., 2025). While single-cell RNA sequencing (scRNA-seq) overcomes this limitation by capturing expression profiles at the single-cell level, the tissue dissociation, cell capture, and library construction processes require cells to be removed from their native environment, preventing the recording of their original spatial coordinates (Luo et al., 2025). Spatial transcriptomics, propelled by advances in *in-situ* capture chemistry, barcoded matrix multiplexing, optical imaging, and high-throughput sequencing, now enables concurrent mapping of gene expression and tissue architecture at single-cell resolution (Rao et al., 2021; Tian et al., 2023). By integrating molecular tagging, precise spatial indexing, and omics readouts, spatial transcriptomics affords an unprecedented view of cellular heterogeneity and spatial organization (Burgess, 2019; Rao et al., 2021; Tian et al., 2023; Wang et al., 2023b). Consequently, spatial transcriptomics has become indispensable for dissecting cell-fate decisions, unraveling mechanisms of tissue morphogenesis, and characterizing the dynamic remodeling of disease microenvironments. In recent years, spatial transcriptomics has advanced rapidly: matrix-capture platforms such as Visium, Slide-seq, and HDST now provide subcellular-resolution, two-dimensional transcriptomic maps, while optical *in-situ* hybridization methods like MERFISH and seqFISH+ use large probe libraries and iterative imaging to approach whole-transcriptome spatial profiling (Burgess, 2019; Wang et al., 2023a; Sun et al., 2025). Building on this progress, technologies including STARmap and Stereo-seq couple single-cell nucleic acid amplification with three-dimensional imaging, greatly increasing sequencing depth and expanding the spatial dimension of analysis (Bawa et al., 2024; Fang et al., 2025). In conclusion, a systematic review of the development, core principles, and applications of spatial transcriptomics in diverse fields like plants and microbiology not only offers novel perspectives for exploring cell fate lineages and organogenesis mechanisms but also lays a theoretical and practical foundation for subsequent technological advancements and interdisciplinary integration.

## 2 Overview of spatial transcriptomics technology

Spatial transcriptomics combines deep transcriptome profiling with microscopy to map gene expression in intact tissues, revealing cell identities, developmental lineages, and regulatory networks beyond the reach of conventional single-cell methods (Longo et al., 2021; Bawa et al., 2024; Zhao et al., 2024b). High-throughput chip-based platforms, such as 10× Visium, Slide-seq V2, Stereo-seq and related technologies, now predominate because they combine sub-cellular resolution, near-complete transcriptome capture and automation, enabling quantitative, spatially explicit analyses of tissue heterogeneity and phenotype–gene associations (**Figure 1**) (Ståhl et al., 2016; Rodriques et al., 2019; Yin et al., 2023; Bawa et al., 2024; Zhao et al., 2024b). By encoding positional barcodes and unique molecular identifiers, these methods yield absolute transcript counts instead of pseudo-temporal inferences alone (Zhao et al., 2024a). However, advancing spatial multi-omics in plants is still constrained by structural and biochemical hurdles: rigid cell walls impede clean cryosectioning, expansive vacuoles dilute intracellular content, and abundant polyphenols inhibit enzymatic reactions, while limited reference genomes hinder precise read mapping (Giacomello and Lundeberg, 2018; Gurazada et al., 2021; Chen et al., 2023; Yin et al., 2023). Overcoming these obstacles will require coordinated advances in sample preparation, reaction chemistry, microfluidic chip engineering, and bioinformatic pipelines to align plant research capabilities with those achieved in animal systems.

## 3 Spatiotemporal transcriptomics technologies: principles and evolution

Spatial transcriptomics has progressed from low-throughput but precise laser-capture microdissection (LCM), to *in situ* hybridization and sequencing that map gene expression in tissue but are limited by probe number and imaging depth, and finally to *in situ* capture with high-throughput sequencing, which preserves spatial coordinates while greatly expanding coverage and resolution (Emmert-Buck et al., 1996; Femino et al., 1998; Ke et al., 2013; Nichterwitz et al., 2016; Ståhl et al., 2016; Chen et al., 2022). While these methodologies have revolutionized our understanding of cellular heterogeneity in mammalian systems, their adaptation to plants lags behind owing to the presence of rigid cell walls, limited probe penetration, and the frequent need for transgenic material in auxiliary techniques such as FACS and INTACT (Deal and Henikoff, 2011; Giacomello and Lundeberg, 2018; Gurazada et al., 2021; Chen et al., 2022). Current plant-focused efforts therefore pursue two parallel objectives: optimizing existing spatial transcriptomic platforms-whether next-generation sequencing-based or imaging-based-for botanical tissues, and applying these refined tools to address fundamental questions in plant development, physiology, and stress responses (Chen et al., 2023; Yin et al., 2023; Serrano et al., 2024). Continued innovation in probe chemistry, tissue processing, and data integration is essential to surmount plant-specific barriers and to unlock the full potential of spatial transcriptomics across the plant kingdom.

### 3.1 Microdissection-based gene expression technologies

Microanatomy-based gene expression technologies employ laser or mechanical microdissection to isolate cells from precisely defined spatial regions within a tissue section (Deal and Henikoff, 2011; Nichterwitz et al., 2016; Luo et al., 2020). By capturing these targeted cells directly, the method preserves the native microenvironmental context while minimizing contamination from neighboring cell types. The harvested material can then be subjected to transcriptomic analyses, enabling high-resolution profiling of gene expression patterns linked to specific histological niches (Deal and Henikoff, 2011; Nichterwitz et al., 2016).

The earliest laser capture microdissection (LCM) laid the foundation for direct cutting of target cells under a microscope using lasers (Espina et al., 2006). Subsequently, researchers prepared tissues into numerous frozen sections and sequenced them separately to obtain regionalized transcriptome data. Tomo-seq further improved quantitative accuracy and spatial resolution by refining the cDNA library construction process. *In vivo* transcriptomics analysis (TIVA) pioneered overcoming *in vitro* limitations by utilizing cell-penetrating peptides to carry photosensitive tags into living cells, capturing mRNA after light activation to achieve spatiotemporal expression analysis of live cells (Lovatt et al., 2014). Geo-seq, combining LCM with single-cell RNA-seq, enables the resolution of transcriptomes in specific regions at the subcellular-level, while NICHE-seq, using GFP labeling and flow cytometry sorting, achieves high-throughput sequencing, though it struggles to resolve precise relative positions between cells, despite locating to specific niches (Chen et al., 2017; Medaglia et al., 2017). ProximID, through gentle dissociation that preserves cell-cell interaction structures, coupled with LCM sorting units, enables single-cell sequencing of local cell interaction environments (Boisset et al., 2018; Asp et al., 2020).

### 3.2 *In-situ* hybridization technologies

In recent years, *in-situ* hybridization (ISH) has progressed rapidly, evolving from rudimentary chromogenic assays to highly sensitive, multiplexed fluorescent platforms that enable precise spatial mapping of nucleic acids within intact tissues (Raj et al., 2008). Early smFISH, limited by probe number, detected only a few genes, though shorter, more numerous probes raised throughput (Raj et al., 2008). SeqFISH then used repeated hybridization–imaging–stripping cycles with binary encoding to broaden transcript detection (Shah et al., 2018). MERFISH followed, adding error-robust codes and combinatorial labeling to improve accuracy and speed (Chen, 2015). Most recently, smHCR and seqFISH+ expanded laser channels and encoding capacity, enabling visualization of tens of thousands of genes in a single experiment (Zhou et al., 2019). Besides barcode-based techniques, osmFISH uses iterative hybridization and direct imaging to quickly survey large tissues (Codeluppi et al., 2018). RNAscope employs paired “Z” probes with signal amplification, achieving single-molecule sensitivity and high specificity while preserving tissue architecture (Wang et al., 2012). DNA microscopy dispenses with optics, inferring molecular positions from ligation frequency data; its resolution is still limited, but it inaugurates a novel paradigm for spatiotemporal transcriptomics (Chang et al., 2006; Weinstein et al., 2019).

### 3.3 *In-situ* sequencing technologies

*In-situ* sequencing is a class of methods for directly detecting and sequencing transcripts at high resolution within the spatial context of cells, which core principle involves signal amplification using DNA nanoballs at the micrometer to nanometer scale, thereby enabling the acquisition of transcriptomic data at the molecular level while preserving tissue structural information (Ke et al., 2013). However, limited by inherent cellular crowding and the resolution of optical systems, this technology has been restricted to analyzing a limited number of transcripts simultaneously (Qian and Lloyd, 2003). Consequently, researchers are continuously developing diverse strategies to overcome this bottleneck (Qian and Lloyd, 2003). In 2013, the first *in-situ* sequencing protocol used padlock probes to capture reverse-transcribed cDNA, amplified it into micrometer-scale rolling circle products (RCPs), and decoded them by sequencing-by-ligation (SBL), laying the groundwork for the field (Ke et al., 2013). Subsequently, BaristaSeq, while retaining padlock probes, significantly improved signal stability and sequencing read length by chemically crosslinking RCPs to the cellular matrix and employing SBS for sequencing (Chen et al., 2018). HybISS, on the other hand, integrated the process into a microfluidic platform for automated operation and replaced SBL with SBH to achieve a higher signal-to-noise ratio; this refinement not only reduced background noise but also enhanced experimental reproducibility (Gyllborg et al., 2020). Another significant advancement, STARmap, directly deployed barcoded padlock probes at the RNA level and added a second primer to replace the traditional reverse transcription step, successfully circumventing the limitations of cDNA synthesis efficiency (Lugmayr et al., 2023). It also utilized secondary hybridization to reduce noise, ultimately generating single-stranded DNA nanoballs via RCA and employing SBL for decoding, thereby balancing sensitivity and specificity (Lugmayr et al., 2023).

### 3.4 *In-situ* capture technologies

*In-situ* capture technology, centered around spatially barcoded primers pre-fixed on tissue sections, achieves localized RNA capture through *in-situ* recognition and hybridization (Miyazu et al., 2010; Amini et al., 2025). Subsequently, the signals are amplified, sequenced *ex situ*, and the three-dimensional spatiotemporal distribution is reconstructed using barcode analysis. Compared to traditional *in-situ* hybridization or *in-situ* sequencing methods, this technology eliminates the need for large-scale fluorescent probe libraries, significantly reducing probe throughput limitations. Simultaneously, it utilizes barcode decoding instead of multiple rounds of fluorescence imaging, avoiding spectral crosstalk and enhancing imaging depth (Miyazu et al., 2010; Amini et al., 2025). Since Ståhl et al. introduced spatial transcriptomics in 2016, the field has advanced from coarse regional analyses to whole-transcriptome quantification within a single tissue section (Ståhl et al., 2016). 10x Genomics’ Visium streamlined workflows and data analysis, enabling large, multi-center studies. To meet the demand for finer detail, NanoString’s GeoMx uses UV-released barcoded probes to reach 10 μm resolution and detect proteins (Hernandez et al., 2022). Slide-seq employs micron-scale barcoded beads with SBL and scRNA-seq for high-throughput profiling, while DBiT-seq “prints” orthogonal barcodes onto tissue, capturing mRNA and proteins in the same pixel-the first spatial multi-omics demonstration (Rodriques et al., 2019). APEX-seq uses APEX2 peroxidase to biotinylate and isolate RNAs from specific compartments in living cells, demonstrating subcellular transcriptome capture, yet its dependence on recombinant expression confines its application to *in vitro* systems (Fazal et al., 2019; Wu et al., 2021). High-definition spatial transcriptomics (HDST) raised barcode density to 2 μm, mapping hundreds of thousands of transcripts with high precision (Vickovic et al., 2019; Rao et al., 2021). Stereo-seq delivers subcellular (~500 nm) resolution across centimeter-scale areas to combine morphology with molecular data (Wei et al., 2022), while Seq-Scope overlays high-density barcodes on an Illumina flow cell to attain sub-micron resolution and uncover organelle-level heterogeneity (Cho et al., 2021; Kim et al., 2025). PIXEL-seq replaces discrete barcodes with continuous polony patterns, enabling the detection of over 1,000 transcripts within a 10 μm² area at 1 μm resolution and markedly enhancing sensitivity (Fu et al., 2022). In parallel, sci-Space merges nuclear barcoding from sci-Plex with sci-RNA-seq, efficiently linking single-cell transcriptomes to their spatial coordinates (Srivatsan et al., 2021; Robles-Remacho et al., 2023).

## 4 Applications of spatiotemporal transcriptomics in plant research

Spatial transcriptomics, with its exceptional spatiotemporal resolution, enables the detailed characterization of plant developmental programs, the identification of rare cell types, and the analysis of stress response networks. Initially challenging to apply directly to plant systems due to the cell wall and vacuole, the technology has been successfully implemented in various plants and organs through systematic optimization of key steps such as tissue fixation, permeabilization, and sectioning. Since Giacomello et al. first constructed a high-throughput plant spatial transcriptome atlas in 2017, the technology has progressed from feasibility validation to broad application across multiple species and tissues (Giacomello et al., 2017), with modified protocols repeatedly validated in systems such as Arabidopsis, lentil, *Lotus japonicas*, and wheat (Geng et al., 2013; Du et al., 2023; Yu et al., 2023; Ye et al., 2024; Li et al., 2025; Zhang et al., 2025). Spatial transcriptomics has not only deepened our understanding of plant development, physiology, and evolutionary mechanisms but also provided a novel molecular perspective and technological platform for crop improvement and precision breeding.

Meiosis, a highly conserved and critical division process during the maturation of sexual reproductive cells, has long been a focal point in plant reproductive and developmental biology research. By precisely isolating maize male reproductive cells at distinct meiotic stages with LCM and analyzing them via scRNA-seq, Nelms and Walbot (2019) systematically connected meiotic cell-cycle dynamics to cellular physiology and developmental differentiation trajectories, laying a robust data foundation for dissecting meiotic regulatory networks (Nelms and Walbot, 2019). Buds, transient structures formed during branch and floral organ development, are governed by intricate signaling pathways; through spatiotemporal transcriptomic profiling of Norway spruce female buds across budding (August), elongation (September) and maturation/dormancy (October) stages, Orozco (2020) pinpointed stage-specific gene-expression loci that drive morphological and functional transitions, thereby unveiling the spatial core regulatory network of bud development (Orozco, 2020). Concurrently, Lieben (2017) conducted a comparative transcriptomic study on poplar leaf buds during dormancy and regrowth, clarifying the distinct expression patterns of various cell types at different developmental stages, providing valuable insights into the molecular basis of bud dormancy and regeneration in woody plants (Lieben, 2017). Recent breakthroughs in single-cell spatial omics technologies have further pushed the resolution limits of plant development research. The first application of Stereo-seq in Arabidopsis leaves (Xia et al., 2022) achieved true single-cell spatiotemporal transcriptome profiling, revealing the divergent spatial developmental trajectories of microtubule cells and guard cells in leaves (Xia et al., 2022). Significantly, Liu et al. (2022) optimized the tissue permeabilization conditions for Stereo-seq using a “two-step method” and successfully constructed high-resolution spatiotemporal transcriptomic maps in the fruit pegs, stems, roots, and hypocotyls of the non-model plant peanut (Liu et al., 2022). Guo et al. used 10x Genomics spatial and single-nucleus transcriptomics to map gene activity in early bamboo shoots, reconstruct developmental trajectories, and identify genes and pathways governing procambium differentiation, intercalary meristem formation, and vascular development, thereby advancing our understanding of bamboo growth and guiding molecular improvement (Guo et al., 2024). Using spatial transcriptomics, Li et al. dissected wheat grains 4–12 days after pollination, identified 10 distinct cell types with 192 marker genes, and, through WGCNA, demonstrated that cell-type-specific highly expressed genes exhibit differential functional enrichments that critically regulate grain development and filling (Li et al., 2025).

## 5 Conclusion

While single-cell transcriptomics reveals cellular heterogeneity, it lacks spatial and tissue-level context. Spatiotemporal transcriptomics overcomes this limitation by simultaneously capturing cellular time-space distribution and gene expression, offering unprecedented insights into development, pathology, and evolution. However, its application in plants lags due to limited reference genomes, structural barriers like cell walls, and incompatibility with animal-based platforms. Common challenges, including balancing resolution and throughput, standardizing sample preparation, algorithmic mining, multi-omics integration, and cost control, remain prominent. With continued advancements in sequencing chemistry, micro-nanofabrication, optical imaging, and artificial intelligence, spatiotemporal omics holds the promise of mapping cellular and even subcellular-level four-dimensional expression atlases, driving profound innovations in precision breeding.

## References

[B1] Amini K. Hejazi S. A. Shinnawy O. (2025). Revealing the invisible dimensions of electrochemical carbon capture technologies through In-situ/operando techniques. Materials Today Energy 50:101870. doi: 10.1016/j.mtener.2025.101870

[B2] Asp M. Bergenstråhle J. Lundeberg J. (2020). Spatially resolved transcriptomes-next generation tools for tissue exploration. BioEssays 42, 1900221. doi: 10.1002/bies.201900221, PMID: 32363691

[B3] Bawa G. Liu Z. Yu X. Tran L.-S. P. Sun X. (2024). Introducing single cell stereo-sequencing technology to transform the plant transcriptome landscape. Trends Plant Sci. 29, 249–265. doi: 10.1016/j.tplants.2023.10.002, PMID: 37914553

[B4] Boisset J.-C. Vivié J. Grün D. Muraro M. J. Lyubimova A. Van Oudenaarden A. (2018). Mapping the physical network of cellular interactions. Nat. Methods 15, 547–553. doi: 10.1038/s41592-018-0009-z, PMID: 29786092

[B5] Burgess D. J. (2019). Spatial transcriptomics coming of age. Nat. Rev. Genet. 20, 317–317. doi: 10.1038/s41576-019-0129-z, PMID: 30980030

[B6] Cao Y. Feng X. Ding B. Huo H. Abdullah M. Hong J.. (2025). Gap-free genome assemblies of two Pyrus bretschneideri cultivars and GWAS analyses identify a CCCH zinc finger protein as a key regulator of stone cell formation in pear fruit. Plant Commun. 6, 101238. doi: 10.1016/j.xplc.2024.101238, PMID: 40071379 PMC11956113

[B7] Cao Y. Hong J. Zhao Y. Li X. Feng X. Wang H.. (2024a). *De novo* gene integration into regulatory networks via interaction with conserved genes in peach. Horticulture Res. 11, uhae252. doi: 10.1093/hr/uhae252, PMID: 39664695 PMC11630308

[B8] Cao Y. Mo W. Li Y. Xiong Y. Wang H. Zhang Y.. (2024b). Functional characterization of NBS-LRR genes reveals an NBS-LRR gene that mediates resistance against Fusarium wilt. BMC Biol. 22, 45. doi: 10.1186/s12915-024-01836-x, PMID: 38408951 PMC10898138

[B9] Chang T. N. Parthasarathy S. Wang T. Gandhi K. Soteropoulos P. (2006). Automated liquid dispensing pin for DNA microarray applications. IEEE Trans. automation Sci. Eng. 3, 187–191. doi: 10.1109/TASE.2006.871481

[B10] Chen K. H. (2015). RNA imaging. spatially resolved, highly multiplexed RNA profiling in single cells. Science 348, 6233. doi: science.aaa6090, PMID: 25858977 10.1126/science.aaa6090 PMC4662681

[B11] Chen C. Ge Y. Lu L. (2023). Opportunities and challenges in the application of single-cell and spatial transcriptomics in plants. Front. Plant Sci. 14, 1185377. doi: 10.3389/fpls.2023.1185377, PMID: 37636094 PMC10453814

[B12] Chen A. Liao S. Cheng M. Ma K. Wu L. Lai Y.. (2022). Spatiotemporal transcriptomic atlas of mouse organogenesis using DNA nanoball-patterned arrays. Cell 185, 1777–1792. doi: 10.1016/j.cell.2022.04.003, PMID: 35512705

[B13] Chen X. Sun Y.-C. Church G. M. Lee J. H. Zador A. M. (2018). Efficient *In-situ* barcode sequencing using padlock probe-based BaristaSeq. Nucleic Acids Res. 46, e22–e22. doi: 10.1093/nar/gkx1206, PMID: 29190363 PMC5829746

[B14] Chen J. Suo S. Tam P. P. L. Han J.-D. J. Peng G. Jing N. (2017). Spatial transcriptomic analysis of cryosectioned tissue samples with Geo-seq. Nat. Protoc. 12, 566–580. doi: 10.1038/nprot.2017.003, PMID: 28207000

[B15] Cho C.-S. Xi J. Si Y. Park S.-R. Hsu J.-E. Kim M.. (2021). Microscopic examination of spatial transcriptome using Seq-Scope. Cell 184, 3559–3572. doi: 10.1016/j.cell.2021.05.010, PMID: 34115981 PMC8238917

[B16] Codeluppi S. Borm L. E. Zeisel A. La Manno G. Van Lunteren J. A. Svensson C. I.. (2018). Spatial organization of the somatosensory cortex revealed by osmFISH. Nat. Methods 15, 932–935. doi: 10.1038/s41592-018-0175-z, PMID: 30377364

[B17] Deal R. B. Henikoff S. (2011). The INTACT method for cell type–specific gene expression and chromatin profiling in Arabidopsis thaliana. Nat. Protoc. 6, 56–68. doi: 10.1038/nprot.2010.175, PMID: 21212783 PMC7219316

[B18] Du K. Jiang S. Chen H. Xia Y. Guo R. Ling A.. (2023). Spatiotemporal miRNA and transcriptomic network dynamically regulate the developmental and senescence processes of poplar leaves. Horticulture Res. 10, uhad186. doi: 10.1093/hr/uhad186, PMID: 37899951 PMC10611553

[B19] Emmert-Buck M. R. Bonner R. F. Smith P. D. Chuaqui R. F. Zhuang Z. Goldstein S. R.. (1996). Laser capture microdissection. Science 274, 998–1001. doi: 10.1126/science.274.5289.998, PMID: 8875945

[B20] Espina V. Wulfkuhle J. D. Calvert V. S. Vanmeter A. Zhou W. Coukos G.. (2006). Laser-capture microdissection. Nat. Protoc. 1, 586–603. doi: 10.1038/nprot.2006.85, PMID: 17406286

[B21] Fang S. Xu M. Cao L. Liu X. Bezulj M. Tan L.. (2025). Stereopy: modeling comparative and spatiotemporal cellular heterogeneity via multi-sample spatial transcriptomics. Nat. Commun. 16, 3741. doi: 10.1038/s41467-025-58079-9, PMID: 40258830 PMC12012134

[B22] Fazal F. M. Han S. Parker K. R. Kaewsapsak P. Xu J. Boettiger A. N.. (2019). Atlas of subcellular RNA localization revealed by APEX-Seq. Cell 178, 473–490. doi: 10.1016/j.cell.2019.05.027, PMID: 31230715 PMC6786773

[B23] Femino A. M. Fay F. S. Fogarty K. Singer R. H. (1998). Visualization of single RNA transcripts *In-situ* . Science 280, 585–590. doi: 10.1126/science.280.5363.585, PMID: 9554849

[B24] Fu X. Sun L. Dong R. Chen J. Y. Silakit R. Condon L. F.. (2022). Polony gels enable amplifiable DNA stamping and spatial transcriptomics of chronic pain. Cell 185, 4621–4633. doi: 10.1016/j.cell.2022.10.021, PMID: 36368323 PMC9691594

[B25] Geng Y. Wu R. Wee C. W. Xie F. Wei X. Chan P. M. Y.. (2013). A spatio-temporal understanding of growth regulation during the salt stress response in Arabidopsis. Plant Cell 25, 2132–2154. doi: 10.1105/tpc.113.112896, PMID: 23898029 PMC3723617

[B26] Giacomello S. Lundeberg J. (2018). Preparation of plant tissue to enable Spatial Transcriptomics profiling using barcoded microarrays. Nat. Protoc. 13, 2425–2446. doi: 10.1038/s41596-018-0046-1, PMID: 30353173

[B27] Giacomello S. Salmén F. Terebieniec B. K. Vickovic S. Navarro J. F. Alexeyenko A.. (2017). Spatially resolved transcriptome profiling in model plant species. Nat. Plants 3, 1–11. doi: 10.1038/nplants.2017.61, PMID: 28481330

[B28] Guo J. Luo D. Chen Y. Li F. Gong J. Yu F.. (2024). Spatiotemporal transcriptome atlas reveals gene regulatory patterns during the organogenesis of the rapid growing bamboo shoots. New Phytol. 244, 1057–1073. doi: 10.1111/nph.20059, PMID: 39140996

[B29] Gurazada S. G. R. Cox K. L. Jr. Czymmek K. J. Meyers B. C. (2021). Space: the final frontier-achieving single-cell, spatially resolved transcriptomics in plants. Emerging Topics Life Sci. 5, 179–188. doi: /TLS20200274, PMID: 33522561 10.1042/ETLS20200274

[B30] Gyllborg D. Langseth C. M. Qian X. Choi E. Salas S. M. Hilscher M. M.. (2020). Hybridization-based *In-situ* sequencing (HybISS) for spatially resolved transcriptomics in human and mouse brain tissue. Nucleic Acids Res. 48, e112–e112. doi: 10.1093/nar/gkaa792, PMID: 32990747 PMC7641728

[B31] Hernandez S. Lazcano R. Serrano A. Powell S. Kostousov L. Mehta J.. (2022). Challenges and opportunities for immunoprofiling using a spatial high-plex technology: the NanoString GeoMx^®^ digital spatial profiler. Front. Oncol. 12, 890410. doi: 10.3389/fonc.2022.890410, PMID: 35847846 PMC9277770

[B32] Jiang L. Li X. Lyu K. Wang H. Li Z. Qi W.. (2025). Rosaceae phylogenomic studies provide insights into the evolution of new genes. Hortic. Plant J. 11, 389–405. doi: 10.1016/j.hpj.2024.02.002

[B33] Jiang L. Lin M. Wang H. Song H. Zhang L. Huang Q.. (2022). Haplotype-resolved genome assembly of Bletilla striata (Thunb.) Reichb. f. to elucidate medicinal value. Plant J. 111, 1340–1353. doi: 10.1111/tpj.15892, PMID: 35785503

[B34] Ke R. Mignardi M. Pacureanu A. Svedlund J. Botling J. Wählby C.. (2013). *In-situ* sequencing for RNA analysis in preserved tissue and cells. Nat. Methods 10, 857–860. doi: 10.1038/nmeth.2563, PMID: 23852452

[B35] Kim Y. Cheng W. Cho C.-S. Hwang Y. Si Y. Park A.. (2025). Seq-Scope: repurposing Illumina sequencing flow cells for high-resolution spatial transcriptomics. Nat. Protoc. 20, 643–689. doi: 10.1038/s41596-024-01065-0, PMID: 39482362 PMC11896753

[B36] Li Y. Jiang L. Mo W. Wang L. Zhang L. Cao Y. (2022). AHLs’ life in plants: Especially their potential roles in responding to Fusarium wilt and repressing the seed oil accumulation. Int. J. Biol. Macromolecules 208, 509–519. doi: 10.1016/j.ijbiomac.2022.03.130, PMID: 35341887

[B37] Li X. Wan Y. Wang D. Li X. Wu J. Xiao J.. (2025). Spatiotemporal transcriptomics reveals key gene regulation for grain yield and quality in wheat. Genome Biol. 26, 93. doi: 10.1186/s13059-025-03569-8, PMID: 40217326 PMC11992740

[B38] Lieben L. (2017). Spatial transcriptomics in plants. Nat. Rev. Genet. 18, 394–394. doi: 10.1038/nrg.2017.41, PMID: 28529334

[B39] Liu C. Li R. Li Y. Lin X. Zhao K. Liu Q.. (2022). Spatiotemporal mapping of gene expression landscapes and developmental trajectories during zebrafish embryogenesis. Dev. Cell 57, 1284–1298. doi: 10.1016/j.devcel.2022.04.009, PMID: 35512701

[B40] Longo S. K. Guo M. G. Ji A. L. Khavari P. A. (2021). Integrating single-cell and spatial transcriptomics to elucidate intercellular tissue dynamics. Nat. Rev. Genet. 22, 627–644. doi: 10.1038/s41576-021-00370-8, PMID: 34145435 PMC9888017

[B41] Lovatt D. Ruble B. K. Lee J. Dueck H. Kim T. K. Fisher S.. (2014). Transcriptome *in vivo* analysis (TIVA) of spatially defined single cells in live tissue. Nat. Methods 11, 190–196. doi: 10.1038/nmeth.2804, PMID: 24412976 PMC3964595

[B42] Lugmayr W. Kotov V. Goessweiner-Mohr N. Wald J. Dimaio F. Marlovits T. C. (2023). StarMap: a user-friendly workflow for Rosetta-driven molecular structure refinement. Nat. Protoc. 18, 239–264. doi: 10.1038/s41596-022-00757-9, PMID: 36323866

[B43] Luo M. Cao Y. Hong J. (2025). Opportunities and challenges in the application of single-cell transcriptomics in plant tissue research. Physiol. Mol. Biol. Plants 31:199–209. doi: 10.1007/s12298-025-01558-6, PMID: 40070535 PMC11890805

[B44] Luo C. Fernie A. R. Yan J. (2020). Single-cell genomics and epigenomics: technologies and applications in plants. Trends Plant Sci. 25, 1030–1040. doi: 10.1016/j.tplants.2020.04.016, PMID: 32532595

[B45] Medaglia C. Giladi A. Stoler-Barak L. De Giovanni M. Salame T. M. Biram A.. (2017). Spatial reconstruction of immune niches by combining photoactivatable reporters and scRNA-seq. Science 358, 1622–1626. doi: 10.1126/science.aao4277, PMID: 29217582 PMC7234837

[B46] Miyazu K. Kawahara D. Ohtake H. Watanabe G. Matsuda T. (2010). Luminal surface design of electrospun small-diameter graft aiming at *In-situ* capture of endothelial progenitor cell. J. Biomed. Materials Res. Part B: Appl. Biomaterials 94, 53–63., PMID: 20524181 10.1002/jbm.b.31623

[B47] Nelms B. Walbot V. (2019). Defining the developmental program leading to meiosis in maize. Science 364, 52–56. doi: 10.1126/science.aav6428, PMID: 30948545

[B48] Nichterwitz S. Chen G. Aguila Benitez J. Yilmaz M. Storvall H. Cao M.. (2016). Laser capture microscopy coupled with Smart-seq2 for precise spatial transcriptomic profiling. Nat. Commun. 7, 12139. doi: 10.1038/ncomms12139, PMID: 27387371 PMC4941116

[B49] Orozco A. (2020). A spatial analysis of Norwegian spruce cone developmental stages.

[B50] Qian X. Lloyd R. V. (2003). Recent developments in signal amplification methods for *In-situ* hybridization. Diagn. Mol. Pathol. 12, 1–13. doi: 10.1097/00019606-200303000-00001, PMID: 12605030

[B51] Raj A. Van Den Bogaard P. Rifkin S. A. Van Oudenaarden A. Tyagi S. (2008). Imaging individual mRNA molecules using multiple singly labeled probes. Nat. Methods 5, 877–879. doi: 10.1038/nmeth.1253, PMID: 18806792 PMC3126653

[B52] Rao A. Barkley D. França G. S. Yanai I. (2021). Exploring tissue architecture using spatial transcriptomics. Nature 596, 211–220. doi: 10.1038/s41586-021-03634-9, PMID: 34381231 PMC8475179

[B53] Robles-Remacho A. Sanchez-Martin R. M. Diaz-Mochon J. J. (2023). Spatial transcriptomics: emerging technologies in tissue gene expression profiling. Analytical Chem. 95, 15450–15460. doi: 10.1021/acs.analchem.3c02029, PMID: 37814884 PMC10603609

[B54] Rodriques S. G. Stickels R. R. Goeva A. Martin C. A. Murray E. Vanderburg C. R.. (2019). Slide-seq: A scalable technology for measuring genome-wide expression at high spatial resolution. Science 363, 1463–1467. doi: 10.1126/science.aaw1219, PMID: 30923225 PMC6927209

[B55] Serrano K. Bezrutczyk M. Goudeau D. Dao T. O’malley R. Malmstrom R. R.. (2024). Spatial co-transcriptomics reveals discrete stages of the arbuscular mycorrhizal symbiosis. Nat. Plants 10, 673–688. doi: 10.1038/s41477-024-01666-3, PMID: 38589485 PMC11035146

[B56] Shah S. Takei Y. Zhou W. Lubeck E. Yun J. Eng C.-H. L.. (2018). Dynamics and spatial genomics of the nascent transcriptome by intron seqFISH. Cell 174, 363–376. doi: 10.1016/j.cell.2018.05.035, PMID: 29887381 PMC6046268

[B57] Srivatsan S. R. Regier M. C. Barkan E. Franks J. M. Packer J. S. Grosjean P.. (2021). Embryo-scale, single-cell spatial transcriptomics. Science 373, 111–117. doi: 10.1126/science.abb9536, PMID: 34210887 PMC9118175

[B58] Ståhl P. L. Salmén F. Vickovic S. Lundmark A. Navarro J. F. Magnusson J.. (2016). Visualization and analysis of gene expression in tissue sections by spatial transcriptomics. Science 353, 78–82. doi: 10.1126/science.aaf2403, PMID: 27365449

[B59] Sun Y. Yu N. Zhang J. Yang B. (2025). Advances in microfluidic single-cell RNA sequencing and spatial transcriptomics. Micromachines 16, 426. doi: 10.3390/mi16040426, PMID: 40283301 PMC12029715

[B60] Tian L. Chen F. Macosko E. Z. (2023). The expanding vistas of spatial transcriptomics. Nat. Biotechnol. 41, 773–782. doi: 10.1038/s41587-022-01448-2, PMID: 36192637 PMC10091579

[B61] Vickovic S. Eraslan G. Salmén F. Klughammer J. Stenbeck L. Schapiro D.. (2019). High-definition spatial transcriptomics for *In-situ* tissue profiling. Nat. Methods 16, 987–990. doi: 10.1038/s41592-019-0548-y, PMID: 31501547 PMC6765407

[B62] Wang F. Flanagan J. Su N. Wang L.-C. Bui S. Nielson A.. (2012). RNAscope: a novel *In-situ* RNA analysis platform for formalin-fixed, paraffin-embedded tissues. J. Mol. diagnostics 14, 22–29. doi: 10.1016/j.jmoldx.2011.08.002, PMID: 22166544 PMC3338343

[B63] Wang Y. Liu B. Zhao G. Lee Y. Buzdin A. Mu X.. (2023b). Spatial transcriptomics: Technologies, applications and experimental considerations. Genomics 115, 110671. doi: 10.1016/j.ygeno.2023.110671, PMID: 37353093 PMC10571167

[B64] Wang Q. Zhi Y. Zi M. Mo Y. Wang Y. Liao Q.. (2023a). Spatially resolved transcriptomics technology facilitates cancer research. Advanced Sci. 10, 2302558. doi: 10.1002/advs.202302558, PMID: 37632718 PMC10602551

[B65] Wei X. Fu S. Li H. Liu Y. Wang S. Feng W.. (2022). Single-cell Stereo-seq reveals induced progenitor cells involved in axolotl brain regeneration. Science 377, eabp9444. doi: 10.1126/science.abp9444, PMID: 36048929

[B66] Weinstein J. A. Regev A. Zhang F. (2019). DNA microscopy: optics-free spatio-genetic imaging by a stand-alone chemical reaction. Cell 178, 229–241. doi: 10.1016/j.cell.2019.05.019, PMID: 31230717 PMC6697087

[B67] Wu E. Guo X. Teng X. Zhang R. Li F. Cui Y.. (2021). Discovery of plasma membrane-associated RNAs through APEX-seq. Cell Biochem. biophysics 79, 905–917. doi: 10.1007/s12013-021-00991-0, PMID: 34028638

[B68] Xia K. Sun H.-X. Li J. Li J. Zhao Y. Chen L.. (2022). The single-cell stereo-seq reveals region-specific cell subtypes and transcriptome profiling in Arabidopsis leaves. Dev. Cell 57, 1299–1310. doi: 10.1016/j.devcel.2022.04.011, PMID: 35512702

[B69] Ye K. Bu F. Zhong L. Dong Z. Ma Z. Tang Z.. (2024). Mapping the molecular landscape of Lotus japonicus nodule organogenesis through spatiotemporal transcriptomics. Nat. Commun. 15, 6387. doi: 10.1038/s41467-024-50737-8, PMID: 39080318 PMC11289483

[B70] Yin R. Xia K. Xu X. (2023). Spatial transcriptomics drives a new era in plant research. Plant J. 116, 1571–1581. doi: 10.1111/tpj.16437, PMID: 37651723

[B71] Yu B. Gao P. Song J. Yang H. Qin L. Yu X.. (2023). Spatiotemporal transcriptomics and metabolic profiling provide insights into gene regulatory networks during lentil seed development. Plant J. 115, 253–274. doi: 10.1111/tpj.16205, PMID: 36965062

[B72] Zhang B. Lam T. K. Y. Chen L. Zhang C. Zhu L. Zhang H.. (2025). Single-cell transcriptomics and time-series metabolite profiling reveal the spatiotemporal regulation of flavonoid biosynthesis genes and phytohormone homeostasis by PAP1 in Arabidopsis. BMC Biol. 23, 191. doi: 10.1186/s12915-025-02297-6, PMID: 40598113 PMC12220432

[B73] Zhao C. Xu Z. Wang X. Tao S. Macdonald W. A. He K.. (2024a). Innovative super-resolution in spatial transcriptomics: a transformer model exploiting histology images and spatial gene expression. Briefings Bioinf. 25, bbae052. doi: 10.1093/bib/bbae052, PMID: 38436557 PMC10939304

[B74] Zhao S. Zhang P. Niu S. Xie J. Liu Y. Liu Y.. (2024b). Targeting nucleotide metabolic pathways in colorectal cancer by integrating scRNA-seq, spatial transcriptome, and bulk RNA-seq data. Funct. Integr. Genomics 24, 72. doi: 10.1007/s10142-024-01356-5, PMID: 38594466 PMC11004054

[B75] Zhou W. Yui M. A. Williams B. A. Yun J. Wold B. J. Cai L.. (2019). Single-cell analysis reveals regulatory gene expression dynamics leading to lineage commitment in early T cell development. Cell Syst. 9, 321–337. doi: 10.1016/j.cels.2019.09.008, PMID: 31629685 PMC6932747

